# Microencapsulation of stearic acid with SiO_2_ shell as phase change material for potential energy storage

**DOI:** 10.1038/s41598-020-71940-9

**Published:** 2020-09-14

**Authors:** Shafiq Ishak, Soumen Mandal, Han-Seung Lee, Jitendra Kumar Singh

**Affiliations:** 1 Department of Architectural Engineering, Hanyang University, 1271 Sa 3-dong, Sangnok-gu, Ansan, 15588 Korea; 2 Intelligent Construction Automation Center, Kyungpook National University, 80, Daehak-ro, Buk-gu, Daegu, 41566 Korea; 3 Innovative Durable Building and Infrastructure Research Center, Department of Architectural Engineering, Hanyang University, 1271 Sa-3-dong, Sangnok-gu, Ansan, 15588 Korea

**Keywords:** Environmental sciences, Chemistry, Energy science and technology, Engineering, Materials science

## Abstract

Stearic acid (SA) is being used as phase change material (PCM) in energy storage applications. In the present study, the microencapsulation of SA with SiO_2_ shell was carried out by sol–gel method. Different amounts of SA (5, 10, 15, 20, 30 and 50 g) were taken against 10 ml of tetraethyl orthosilicate (TEOS) for encapsulation. The synthesized microencapsulated PCM (MEPCM) were characterized by Fourier transform infrared spectroscope (FT-IR), X-Ray diffraction (XRD), X-Ray photoelectron spectroscopy (XPS) and scanning electron microscopy (SEM). The characterization results showed that SA was successfully encapsulated by SiO_2_. Thermogravimetric analysis (TGA) exhibited better thermal stability of the MEPCM than SA. The enthalpy values of MEPCM were found to be unchanged even after 30 heating–cooling cycles by differential scanning calorimetry (DSC). The latent heats of melting and solidification of 50 g SA containing MEPCM were found to be highest i.e. 182.53 J/g and 160.12 J/g, respectively among all microencapsulated samples. The encapsulation efficiency values were calculated using thermal data and the efficiency was found to be highest i.e. 86.68% in the same sample.

## Introduction

Approximately 58% of the total energy is being used in the construction sector for heating and cooling of buildings^1^. Therefore, it is utmost required to produce efficient energy system considering environmental pollution^2^. Latent heat technology using phase change materials (PCMs) can store high energy with low-temperature swing^3–6^ which may have wide applications in heat transfer, solar energy storage, aerospace engineering and air-condition^7–9^. PCMs can absorb heat energy from the outer surface of the building in day time while at the night time, the energy can be released^10^. Thus, PCMs are recommended as thermal storage energy materials. Moreover, there are different types of PCMs such as solid–solid, solid–liquid, liquid–gas and solid–gas^11^. Among them, the most popular and commonly used PCMs are solid–solid and solid–liquid. However, it is very difficult to make the application of liquid–gas and solid–gas PCMs owing to their tremendous volume shift.

PCMs have different applications owing to their characteristics which melt below 15 °C may be used in air-conditioning to maintain the coldness, as well as, the one which melts above 90 °C can be used for application in heat to prevent ignition^12^. Depending upon the applications and melting temperature range; different PCMs have been synthesized from different organic and inorganic chemicals^13–15^. Paraffin is the most commonly used PCMs which possesses high latent heat, non-corrosive, safe and wide range of melting point^16–21^.

However, due to low thermal conductivity, PCMs need to be encapsulated with shell (outer layer) to prevent the leakage of core materials during the phase change process^22^. Moreover, there is a possibility that handling error or external pressure can break the outer layer (shell) and the melted PCMs can react with the building materials that can result in corrosion of embedded steel rebar thereby reducing building serviceability^23^. Thus, the synthesis of encapsulated PCMs with an adequate shell material is important which could solve the aforementioned problems^24^.

Microencapsulation of PCMs can efficiently increase the transfer of heat and decrease the reactivity of the external environment as well as can control the volume changes. Different methods have been developed for the encapsulation of PCMs viz. interfacial polymerization^25–28^, in situ polymerization^29–32^, coacervation^33–35^, and sol–gel process^36–39^. Formaldehyde resin can be used for microencapsulation^40–43^. Use of melamine–formaldehyde and urea–formaldehyde resins as shell materials usually release poisonous formaldehyde during application. Thus, these materials are prohibited to be used for encapsulation process. However, the environmental friendly PCM for scalable thermal energy storage can be synthesized by fatty acid-lignin based hybrid nanocapsules ^44^.

Zhang et al. ^45^ have synthesized lauric acid with tetraethylorthosilicate and concluded that, as the volume ratio of methyltriethoxysilane to tetraethylorthosilicate was increased, the latent heat decreased as well as the surface hydrophobicity increased. Lauric acid could be a potential and efficient core material into kapok fiber^46^. Moreover, Latibari et al.^47^ have synthesized stearic acid based PCM where TiO_2_ was used as a shell material. *N*-octadecane and organosilica nanocapsules as potential PCM were prepared by Zhu et al.^48^. From the literature review, it is very difficult to understand about the recommended amount of PCMs used to form an efficient and stable microencapsulated PCMs.

Therefore, to the best of author’s knowledge, the amount of PCMs used for the microencapsulation is a vital parameter to produce efficient and stable microencapsulated PCMs. Different amount of PCMs used will elucidate different characteristics and stability of the microencapsulated PCMs. Stearic acid (fatty acid) is an eco-friendly, medically important and economical which can be used in thermal energy storage as it is having high enthalpy value (~ 200 J/g) and can sustain up to 72 °C. Besides, SiO_2_ is non-inflammable, can provide higher mechanical strength, thermal conductivity, and better chemical resistance to the core materials as well as acts as pozzolanic materials in building applications. During mixing of cement with water, the poorly encapsulated PCM can be broken owing to mechanical attrition and high temperature produced (heat of hydration) in the mass concrete structures. Thus, the alternative of using microencapsulated SA with SiO_2_ shell can solve this problem. Therefore, this research objective is to examine the properties and effect of PCM synthesized via sol–gel process for building application. In the present study, we have systematically studied and encapsulated the SA (as core material) in varying amount of 5, 10, 15, 20, 30 and 50 g with SiO_2_ shell. A fixed amount i.e. 10 ml of tetraethyl orthosilicate (TEOS) was used as the precursor solution to form SiO_2_ shell.

## Experimental

### Materials

Reagent grade stearic acid (SA, C_18_H_36_O_2_, melting point: 72 °C) as core material was obtained from Daejung Chemical & Metals Co., Ltd., Gyeonggi-do, South Korea. Tetraethyl orthosilicate (TEOS, C_8_H_20_O_4_Si) as the precursor solution was obtained from Acros Organics, Geel, Belgium. In addition, anhydrous ethyl alcohol (EA, C_2_H_5_OH) and sodium lauryl sulphate (SLS, C_12_H_25_NaO_4_S) were obtained from Daejung Chemical & Metals Co., Ltd., Gyeonggi-do, South Korea and were used as solvent and surfactant, respectively. Distilled water has also been used as a solvent.

### Preparation of emulsion

Different amount of SA was mixed in 100 ml distilled water with various proportion of sodium lauryl sulphate (SLS) using a magnetic stirrer at 800 rpm and 75 °C for 1 h (Table 1). SA emulsions were prepared in two different sets; (1) SA of 5, 10, and 15 g were mixed with 0.10 g of SLS in 100 ml distilled water (SATEOS1, SATEOS2 and SATEOS3), (2) 20, 30 and 50 g SA with 0.15, 0.20 and 0.25 g SLS were mixed in 100 ml distilled water (SATEOS4, SATEOS5 and SATEOS6). A 0.10 g SLS was used with 5, 10, and 15 g SA to form a proper emulsion. Subsequently, it was required to increase the amount of SLS for SATEOS4, SATEOS5 and SATEOS6. Table 1 shows the proportion of SA and SLS used to produce stable emulsion solution.

**Table 1:** The composition of SA emulsion.

Samples	Stearic acid emulsion		
	Stearic acid (g)	Distilled water (ml)	SLS (g)
SATEOS1	5	100	0.10
SATEOS2	10		
SATEOS3	15		
SATEOS4	20		0.15
SATEOS5	30		0.20
SATEOS6	50		0.25

### Preparation of microencapsulated stearic acid with SiO_2_ shell

**Figure 1:**
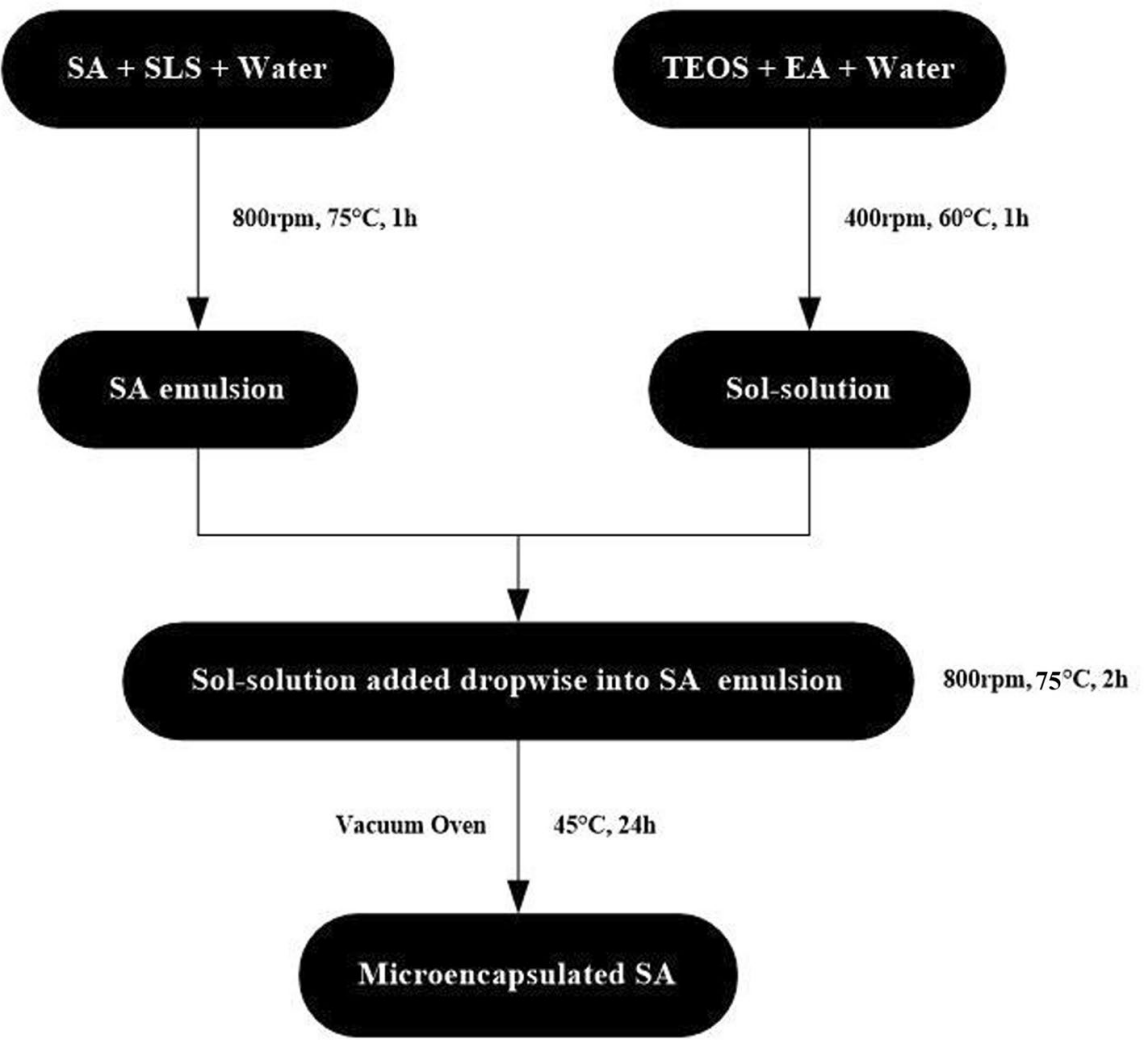
Schematic for preparation of microencapsulated SA.

10 ml TEOS, 10 ml ethyl alcohol (EA) and 20 ml distilled water were taken into 100 ml beaker. The ratio was fixed for the synthesis of all samples to investigate the encapsulation efficiency on different percentage of SA with SiO_2_ shell. The mixture was stirred with magnetic stirrer at 400 rpm and 60 °C for 1 h. The precursor solution was then added dropwise to the prepared SA emulsion under vigorous stirring at 800 rpm and 75 °C for 2 h which resulted into white powders after filtration. This white powder was washed with distilled water to remove the residual SA and was dried in a vacuum oven at 45 °C for 24 h. Ultimately, microencapsulated SA with SiO_2_ shell was obtained. The overall process of synthesis and preparation of microencapsulated SA is described schematically in Fig. 1.

**Figure 2:**
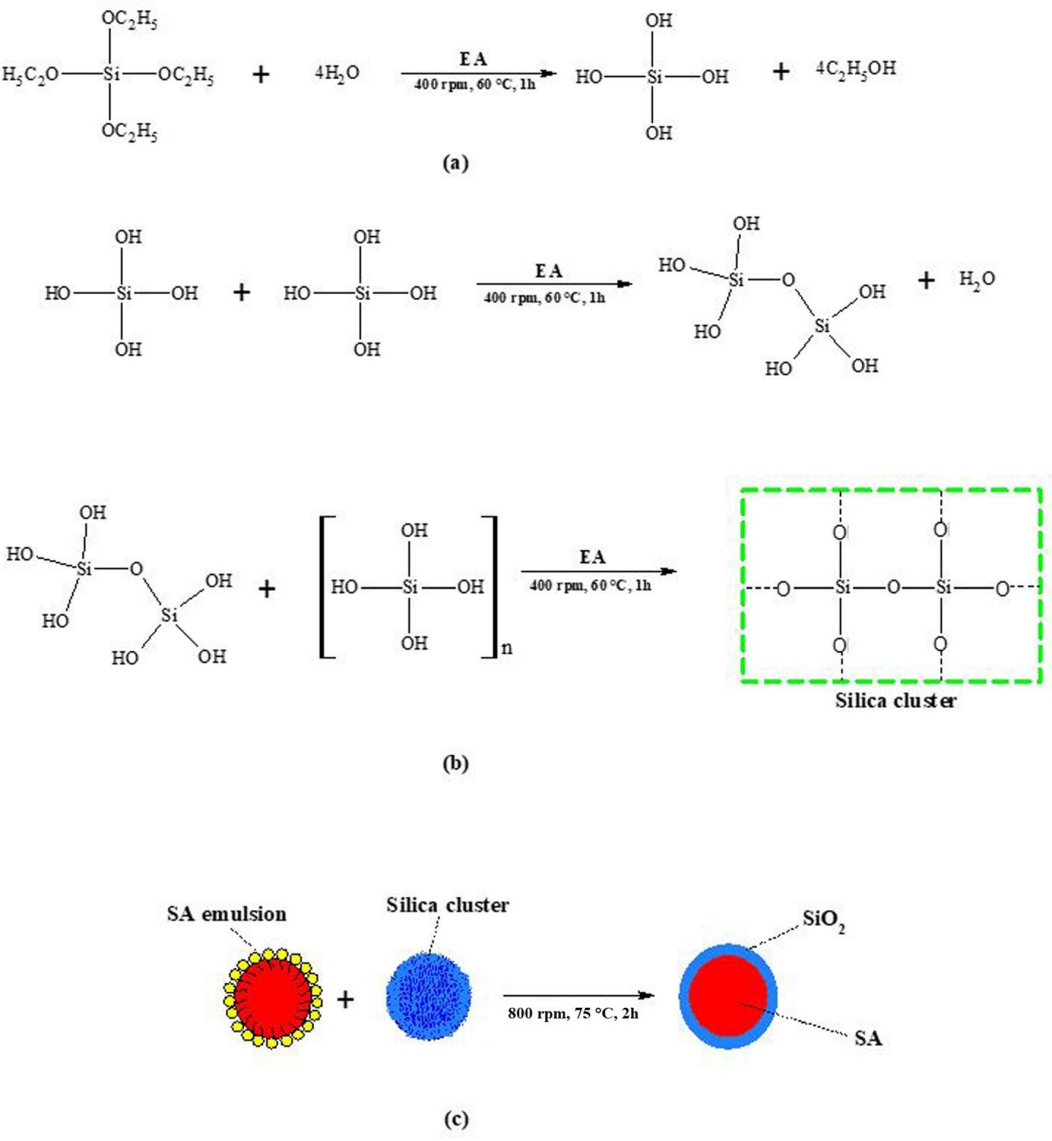
Schematic for microencapsulation of SA with SiO_2_ shell **(a)** hydrolysis of TEOS **(b)** condensation of hydrolysed product and **(c)** encapsulation of SA with SiO_2_ shell.

Microencapsulated SA with SiO_2_ shell was prepared through sol–gel method and the mechanism of the encapsulation is described in Fig. 2. The first step involves the preparation of SA emulsion in an aqueous solution with the presence of SLS as surfactant. In this case, the hydrophobic end of SA molecules was bound with SLS while the hydrophilic end was bound with water molecule, resulted in the formation of stable emulsion. Therefore, the hydrophobic SLS segments were protected and covered the SA droplet surface. On the other hand, the hydrolysis of TEOS solution occurred slowly with water molecule resulting into the formation of hydrolysed TEOS (Fig. 2a) in presence of ethyl alcohol^49–51^. The hydrolysed TEOS was proceeded for condensation reaction where n number of hydrolysed TEOS would form cluster of silica (Fig. 2b). This cluster of silica encapsulates the SA^52^ in the presence of SLS (Fig. 2c) which known as microencapsulation process.

### Characterization techniques

The chemical analysis of bulk SA and microencapsulated SA was performed by Fourier transform infrared spectroscope (FT-IR, Perkin Elmer UATR Two, United States of America) and the spectra were recorded from 500 to 4,000 cm^−1^.

X-ray diffractometer (XRD, D/MAX-2500, Rigaku, Japan) was used to analyse the phases of bulk SA and microencapsulated materials. XRD scans were carried out from 2θ = 5°–95° at 4°/min scan rate with Cu-K_α_ radiation (λ = 1.541 Å), 25 kV and 100 mA operating conditions on continuous scanning mode. The XRD scans were plotted from 2θ = 5°–50° because there is no peak observed after 50º in all samples.

X-ray photoelectron spectroscopy (XPS, Scienta Omicron R3000, United States of America) was performed with Al *K*_α_ (1,486.6 eV) as source of X-ray radiation to understand the chemical states of the elements present in bulk SA as well as encapsulated materials. The collected XPS spectra were calibrated with adventitious carbon (284.6 eV binding energy) for C 1*s* peak. The high resolution peaks for individual elements were deconvoluted and fitted with Gaussian/Lorentzian function using CASA XPS software after the background correction using Shirley method.

The morphology of bulk SA and microencapsulated SA were examined by a scanning electron microscopy (SEM, MIRA3, TESCAN, Brno, Czech Republic) equipped with energy-dispersive X-ray spectroscopy (EDS) at 15 kV. Prior to take the SEM images, the samples were coated with platinum (Pt) to avoid charging effect.

The thermal properties (melting/solidification temperature and latent heat) and reliability (thermal cycle) were performed by differential scanning calorimetry (DSC, TA Instrument, Discovery DSC, New Castle, USA) at 10 °C/min heating/cooling rate between 40 and 90 °C under continuous purging of nitrogen. The weight loss analysis was conducted by TGA analyser (TA Instrument, Discovery TGA, New Castle, USA) at a heating rate of 10 °C/min starting from 40–600 °C under continuous flow of nitrogen.

## Results and discussion

### FT-IR analysis

**Figure 3:**
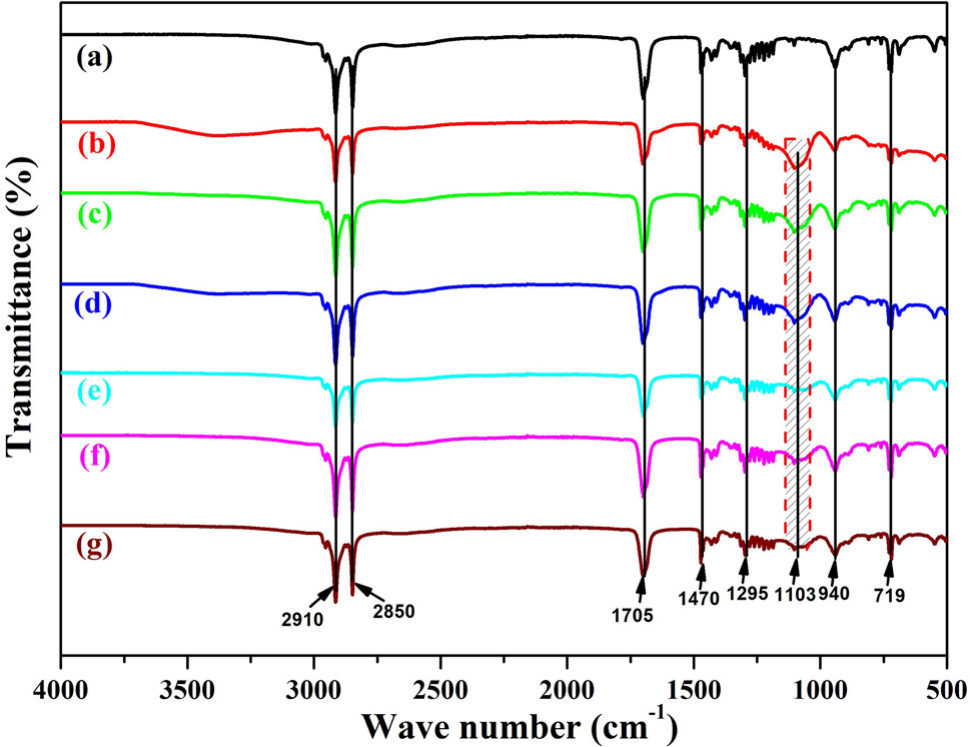
FT-IR spectra of the **(a)** SA, **(b)** SATEOS1, **(c)** SATEOS2, **(d)** SATEOS3, **(e)** SATEOS4, **(f)** SATEOS5 and **(g)** SATEOS6.

Figure 3 demonstrates the FT-IR spectra of bulk SA as well as microencapsulated SA (SATEOS1, SATEOS2, SATEOS3, SATEOS4, SATEOS5, and SATEOS6). The absorption peaks at 2,910 cm^−1^ and 2,850 cm^−1^ in all samples (SA as well as microencapsulated SA) attributed to the symmetrical stretching vibration of –CH_3_ and –CH_2_ groups, respectively^10,50^. The peak at 1705 cm^−1^ corresponds to the vibrational stretching of C=O bond. The peaks at 1,470 cm^−1^ and 1,295 cm^−1^ assigned to –OH functional group’s in-plane bending vibrations whereas, at 940 cm^−1^ and 719 cm^−1^ corresponds to in-plane swinging vibration and out-of-plane bending vibration of the –OH group, respectively. The absorption peaks of SA at 2,910, 2,850, 1,705, 1,470, 1,295, 940 and 719 cm^−1^ also appeared in all microencapsulated SA. Apart from that, newly discovered peak at 1,103 cm^−1^ is found in microencapsulated SA which corresponds to anti-symmetrical stretching vibration of Si–O–Si band. The finding of FT-IR results corroborates with the result of Yuan et al.^50^ where they have successfully fabricated the microencapsulated SA with ammonia-to-ethyl alcohol ratio and found that there is no chemical interaction of SA with SiO_2_. FT-IR results of the current study suggest that SiO_2_ shell has successfully encapsulated the SA (core) through the condensation and polymerization process of hydrolysed TEOS. The peaks intensity of Si–O–Si band is higher at lower amount of SA (Fig. 3b–d). As the amount of SA is increased above 15 g, the peak intensity and broadening of Si–O–Si band decreased gradually which infer that thin layer of SiO_2_ has been formed on the surface of SA.

### XRD analysis

**Figure 4:**
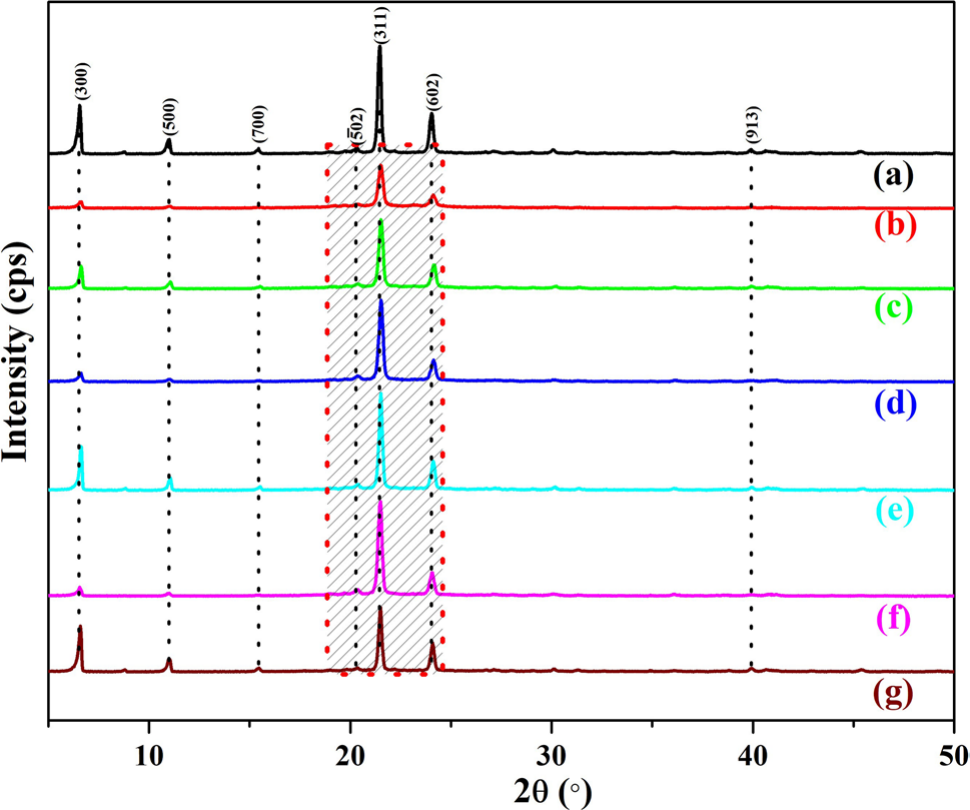
XRD patterns of **(a)** SA, **(b)** SATEOS1, **(c)** SATEOS2, **(d)** SATEOS3, **(e)** SATEOS4, **(f)** SATEOS5 and **(g)** SATEOS6.

The XRD patterns of bulk SA and microencapsulated SA are shown in Fig. 4. The XRD peaks at 2θ = 6.50° (300), 10.94° (500), 15.46° (700), 20.26° $(\overline{5}02)$, 21.42° (311), 24.04° (602) and 39.98° (913) in all samples are assigned to SA according to JCPDS No. 038­1923. There is a little shift in 2θ value of the microencapsulated SA compared to bulk SA owing to the distortion and heterozygosity caused by some uncertain factor such as surfactant (SLS), other residual substances and microencapsulation with SiO_2_^50^. Once the encapsulation has occurred, the intensity of the main peaks at (300), (500), (311) and (602) is gradually decreased compared to bulk SA which indicates that the crystallinity of the sample is decreased.

SATEOS1 shows sharp decrease in intensity compared to other samples. There is no other peak observed in all microencapsulated samples (Fig. 4b–g) which confirms that there is no chemical interaction occurred rather than physical adsorption of SiO_2_ on SA surface^52^. Besides, it also concludes that microencapsulated SA does not involve in occurrence of any new structure. SiO_2_ remains intact on the surface of SA without any chemical reaction, and the existing peaks become more and more obvious with low amount of SA used (SATEOS1). This result suggests that SiO_2_ encapsulates the surface of SA mostly. The peak at (700) is completely disappeared while peak at $(\overline{5}02)$ becomes a hump in SATEOS 1 (Fig. 4b) which is attributed to the decrease in crystallinity and increase in amorphousity. SiO_2_ is amorphous in nature thus, there are hump and broadening^53^ in peaks observed from 2θ = 19° to 25° (Fig. 4b–g) which confirms the presence of amorphous SiO_2_^52^. The lower in diffraction peaks intensity of microencapsulated SA is attributed to the nucleation effect of the inner silica wall and confined behaviour of crystallization^49^. It is believed that at lower SA content, thicker silica shell would form owing to the presence of high amount of TEOS, which is significantly adsorbed on the outer surface of SA. But once the SA amount is increased, the surface area of SA droplet in the emulsion solution increases where higher amount of TEOS is required for proper encapsulation. Thus, at higher amount of SA, the SiO_2_ peak is suppressed in FT-IR (Fig. 3) and the diffraction peak intensity around 2θ = 19°–25° in XRD (Fig. 4) is decreased as well as the broadening is not observed. However, it can be seen from Fig. 4 that once the SA amount is increased from 5 g (SATEOS1) to 50 g (SATEOS6), the peaks shifted very closely to the bulk SA as well as the peak at (700) appeared with all well-established peak intensity. This finding correlates with FT-IR results where SiO_2_ peak intensity is decreased at 1,103 cm^−1^ for SATEOS6 (Fig. 3g).

### XPS analysis

**Figure 5:**
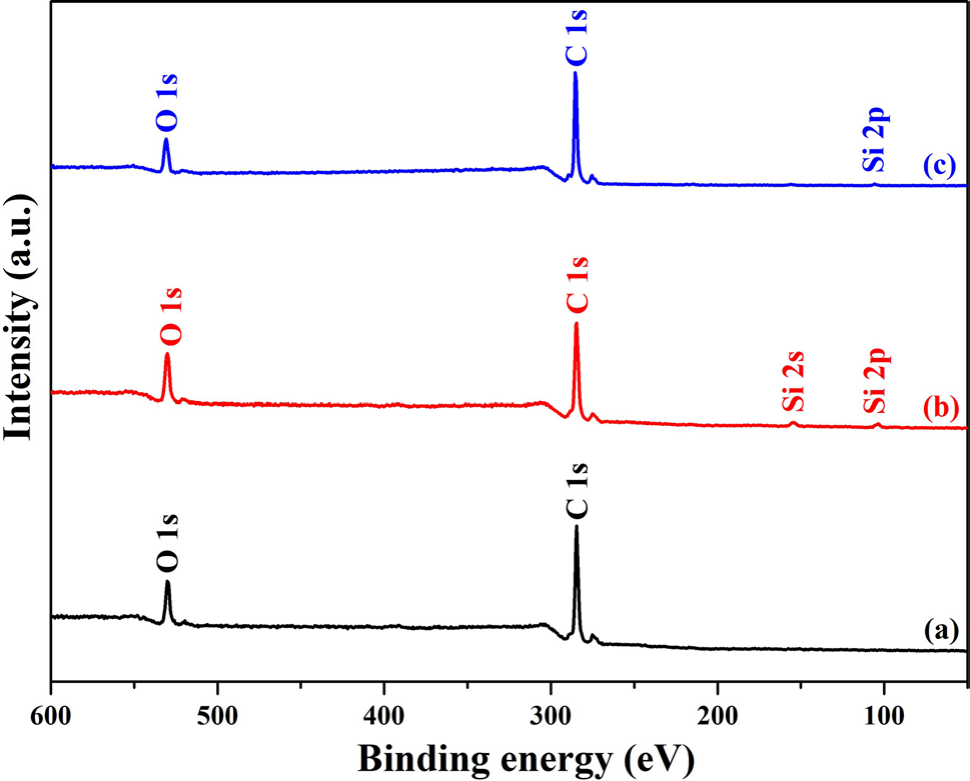
XPS survey scan of **(a)** SA, **(b)** SATEOS1 and **(c)** SATEOS6.

**Figure 6:**
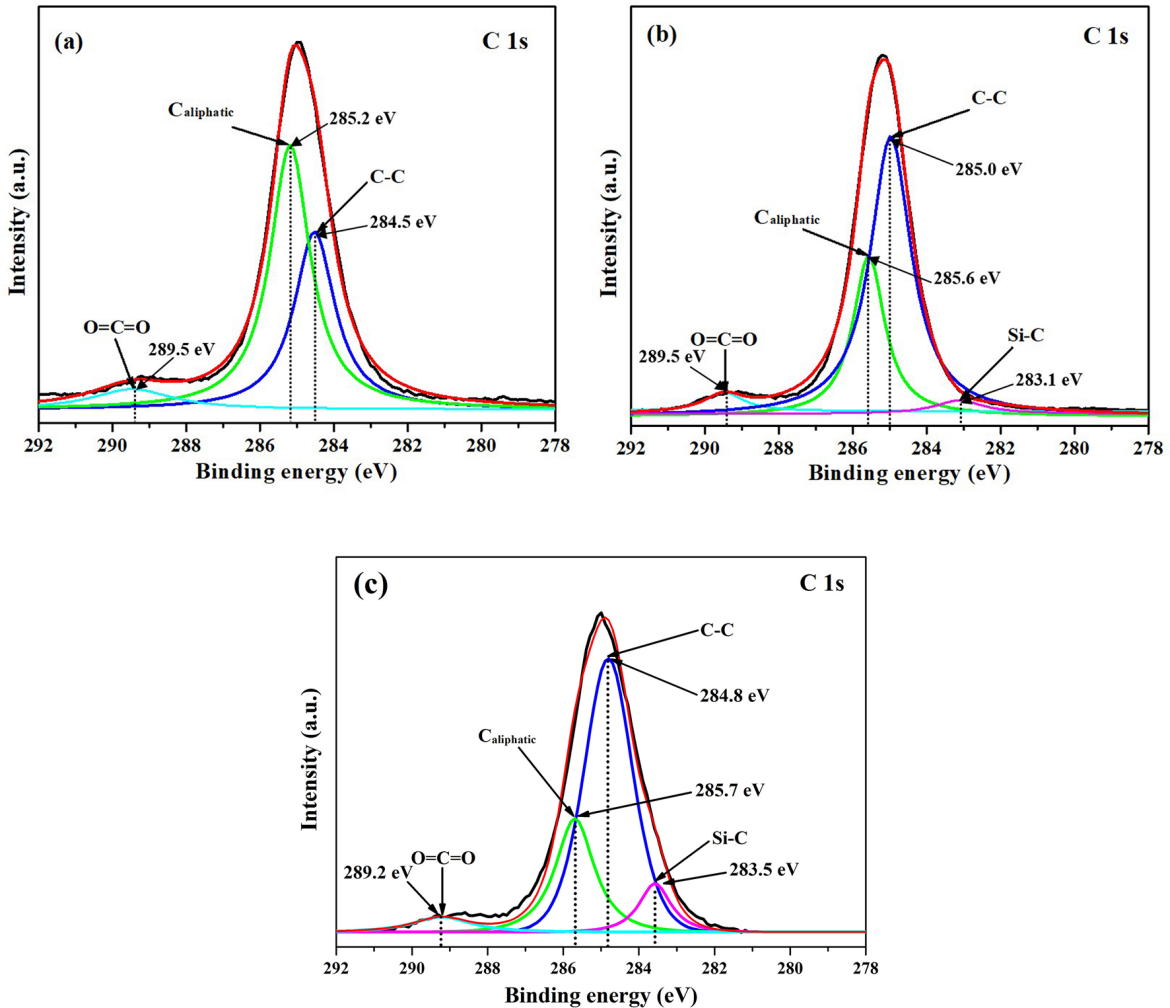
XPS spectra of C 1*s* of **(a)** SA, **(b)** SATEOS1 and **(c)** SATEOS6.

**Figure 7:**
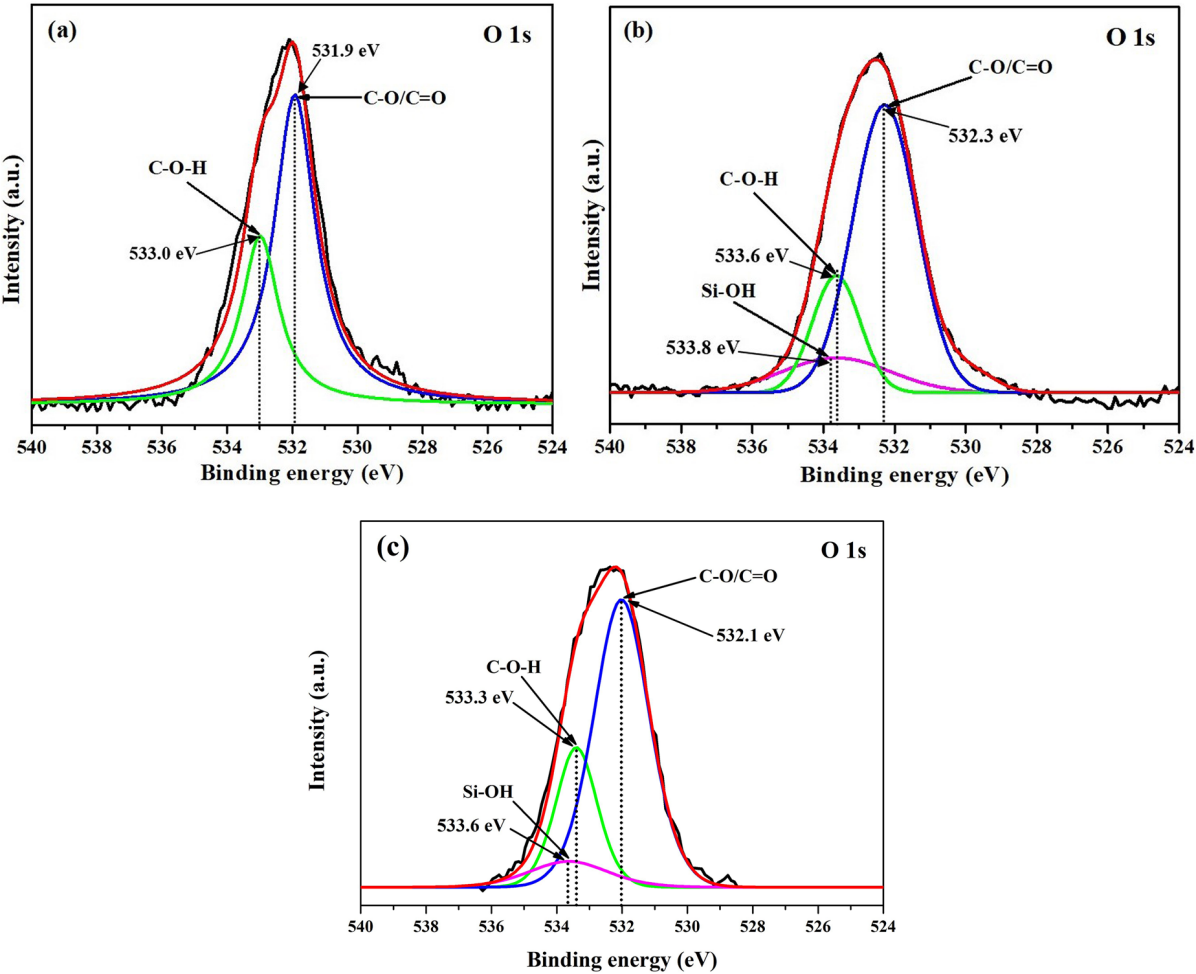
XPS spectra of O 1*s* of **(a)** SA, **(b)** SATEOS1 and **(c)** SATEOS6.

**Figure 8:**
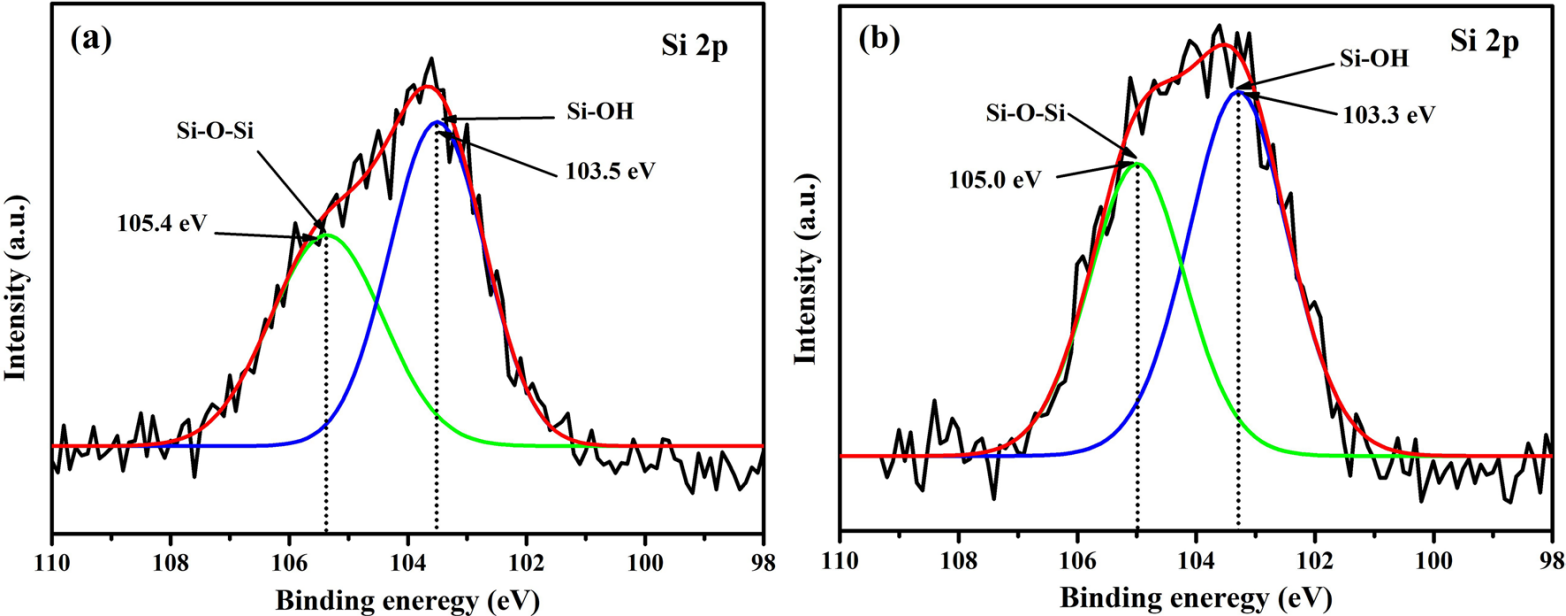
XPS spectra of Si 2*p* of **(a)** SATEOS1 and **(b)** SATEOS6.

The chemical states of the elements present in SA, SATEOS1 and SATEOS6 are shown in Figs. 5, 6, 7 and 8 and Table 2. The survey scans of bulk SA, SATEOS1 and SATEOS6 are shown in Fig. 5 whereas the high resolution scans for C 1*s*, O 1*s* and Si 2*p* are presented in Figs. 6, 7 and 8, respectively. The binding energy values obtained by XPS are summarized in Table 2. It can be seen in Fig. 5 that, distinct peaks of Si 2*s* and Si 2*p* are observed in SATEOS1 and SATEOS6 where microencapsulation with SiO_2_ shell is occurred. The similar Si 2*s* peak at 155.1 eV has been reported by the previous researchers^54^. The presence of Si peaks in SATEOS1 (Fig. 5b) and SATEOS6 (Fig. 5c) corroborate with the findings of FT-IR (Fig. 3) and XRD (Fig. 4).

**Table 2:** Binding energy (eV) of SA, SATEOS1 and SATEOS6.

Peak	Elements	Binding energy (eV)			References
		SA	SATEOS1	SATEOS6	
C 1*s*	C–C	284.5	285.0	284.8	284.8^55^
	C_aliphatic_	285.2	285.6	285.7	285.6^55^
	O=C=O	289.5	289.5	289.2	289.7^55^
	Si–C	–	283.1	283.5	283.2^56^
O 1*s*	C–O–H	533.0	533.6	533.3	533.1^57,58^
	C–O/C=O	531.9	532.3	532.1	532.4^55,57^
	Si–OH	–	533.8	533.6	533.2^55^
Si 2*p*	Si–O–Si	–	105.4	105.0	104.9^55^
	Si–OH	–	103.5	103.3	103.7^55^

As shown in Fig. 6a, C 1*s* of bulk SA is fitted with three different peaks for C–C, C_aliphatic_ and O=C=O at 284.5 eV, 285.2 eV and 289.5 eV binding energy, respectively. The C–C, C_aliphatic_ and O=C=O peaks are also observed in SATEOS1 (Fig. 6b) and SATEOS6 (Fig. 6c) as well as summarised in Table 2. Apart from that, the C 1*s* peak is also fitted with one extra peak of Si–C at 283.1 eV (SATEOS1) and 283.5 eV (SATEOS6). Our observed binding energies of C–C, C_aliphatic_, O=C=O and Si–C well correlates with the other references^55,56^.

The XPS spectra of O 1*s* for bulk SA, SATEOS1 and SATEOS6 are shown in Fig. 7a–c, respectively. The O 1*s* peak of bulk SA is deconvoluted and fitted with two peaks which are C=O/C–O (531.9 eV) and C–O–H (533.0 eV) whereas O 1s of SATEOS1 and SATEOS6 are fitted with three peaks which are C=O/C–O, C–O–H and Si–OH^55,57,58^. There is slight shifting in binding energy of O 1*s* in SATEOS1 and SATEOS6 compared to bulk SA owing to the change in chemical moiety due to the presence of SiO_2_ and Si–OH in the shell material.

The XPS spectra of Si 2*p* for SATEOS1 and SATEOS6 are illustrated in Fig. 8a,b, respectively. No Si 2*p* is observed in bulk SA owing to the absence of SiO_2_. The Si 2*p* peak is fitted at 105.4 eV for SATEOS1 and 105.0 eV for SATEOS6 correspond to Si–O–Si whereas peak at 103.5 eV for SATEOS1 and 103.3 eV for SATEOS6 correspond to Si–OH^55^. The fitting of Si–O–Si and Si–OH peaks in SATEOS1 and SATEOS6 reveal the successful microencapsulation of SiO_2_ on the surface of core SA.

### Morphology of the microencapsulated SA with SiO_2_ shell

**Figure 9:**
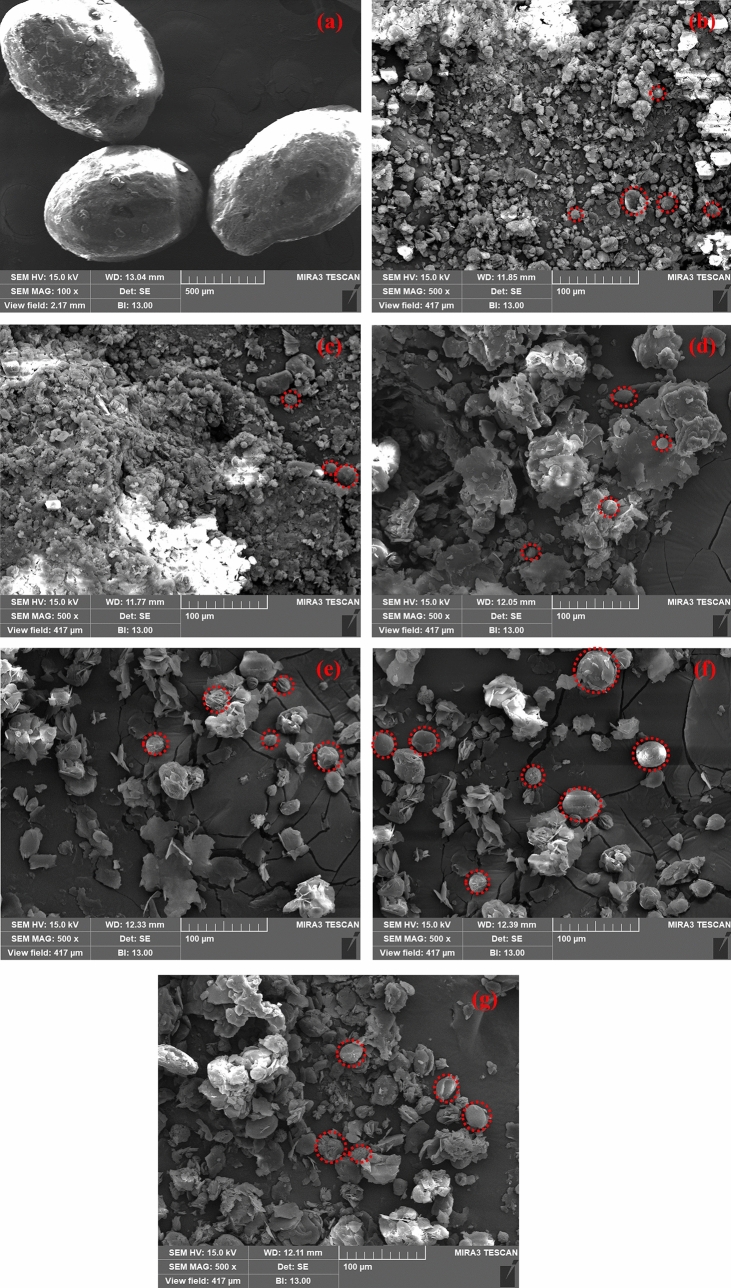
SEM photographs of **(a)** SA (at ×100), **(b)** SATEOS1, **(c)** SATEOS2, **(d)** SATEOS3, **(e)** SATEOS4, **(f)** SATEOS5 and **(g)** SATEOS6 at ×500.

The morphology of microencapsulated materials is very important which influences the solubility, stability, chemical reactivity, flowability and strength^59^. Therefore, SEM has been used to characterise the morphology of bulk SA (at 100×) as well as microencapsulated SA (at 500×), as shown in Fig. 9. It can be seen from Fig. 9a that bulk SA exhibits oval shape with more than 500 µm particles size. However, once the microencapsulation process was performed, the morphology has changed dramatically as shown in Fig. 9b–g.

SATEOS1 sample exhibits smaller quasi-spherical SA particles encapsulated by SiO_2_ with coarse surface (Fig. 9b) which might be owing to the rapid diffusion of ethyl alcohol molecules accelerated by the hydrolysis and polycondensation of TEOS on the surface of the SA. Therefore, SiO_2_ particles is deposited and agglomeration is observed^52,60^. This SiO_2_ shell provides mechanical strength to the microencapsulated SA particles which as well prevent from the leakage of melted SA at higher temperature^10^. This result suggests that microencapsulated SA with SiO_2_ acts as potential energy storage material^61^. From Fig. 9b, it can be seen that SATEOS1 sample has uniform distribution of particles where thick layer of SiO_2_ encapsulates the SA. The particle size of microencapsulated SA (SATEOS1) is found to be around 10–20 µm (Fig. 9b) that is much smaller compared to bulk SA attributed to the lower amount of SA. The thickness of microencapsulation layer is caused by the hydrolysis and polycondensation of the precursor solution. The conglomeration has occurred in lower amount of SA i.e. up to 15 g (Fig. 9b–d) but once the amount is increased, no agglomeration is observed rather than well-defined globular particles (Fig. 9e–g)^62^.

Besides, there is influence of SA content (SATEOS1, SATEOS2 and SATEOS3) with constant amount of surfactant i.e. SLS on the efficiency, shape and particles size distribution. Therefore, it is observed that SATEOS1 shows smaller particles size, uniform distribution and compact surface (Fig. 9b) attributed to the hydrophilicity of SA which favour secondary nucleation at constant surfactant^63^. It is believed that by increasing the SA content from 5 to 15 g (SATEOS1, SATEOS2 and SATEOS3) with constant amount of surfactant i.e. 0.10 g SLS (Table 1), the contribution of surfactant molecule per particle would decrease thus, the particles size and distribution of SATEOS2 (Fig. 9c) and SATEOS3 (Fig. 9d) is differed compared to SATEOS 1 (Fig. 9b).

SATEOS2 shows the dense morphology of microencapsulated SA as well as the particle size is increased (Fig. 9c) compared to SATEOS1 (Fig. 9b). It is attributed to the agglomeration where the condensation rate (Fig. 2b) is decreased^49^. As the amount of SA is increased with SLS, the microencapsulation is clearly visible as observed in Fig. 9e–g attributed to the lesser amount of silica oligomers (from TEOS) which is able to deposit and form well-defined microcapsule on emulsion of SA rather than the occurrence of agglomeration. Moreover, it is observed from Fig. 9e–g that all particles are well-defined with globular in shape and size. It is realized that in the presence of high amount of SA, proper amount of silica oligomers is available which causes proper condensation and encapsulation, therefore, well-defined microcapsules are formed^49^. It is depicted from SEM results that SATEOS6 form proper microcapsule compared to low amount of SA.

The Energy Disperse X-Ray Spectroscopy (EDS) results of bulk SA and microencapsulated SA are shown in Table 3. From this table, it is observed that the amount of Si gradually decreases from SATEOS1 (12.34%) to SATEOS6 (2.68%) with the increased amount of SA. Therefore, it can be said that increase in amount of SA causes in the reduction of SiO_2_ deposition on the surface of SA. There is no consistent value in the amount of C and O shown in Table 3 which is attributed to the semi-quantitative analysis of EDS^51^. The amount of Si in the microencapsulated SA correlates with the FT-IR, XRD and XPS results.

**Table 3:**
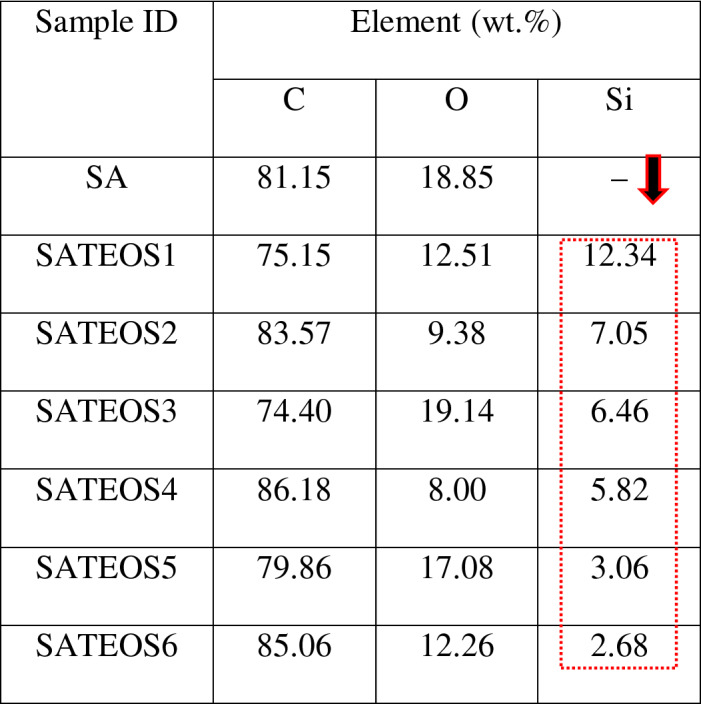
EDS result of SA and microencapsulated SA.

### Thermal properties of the microencapsulated SA with SiO_2_ shell

**Figure 10:**
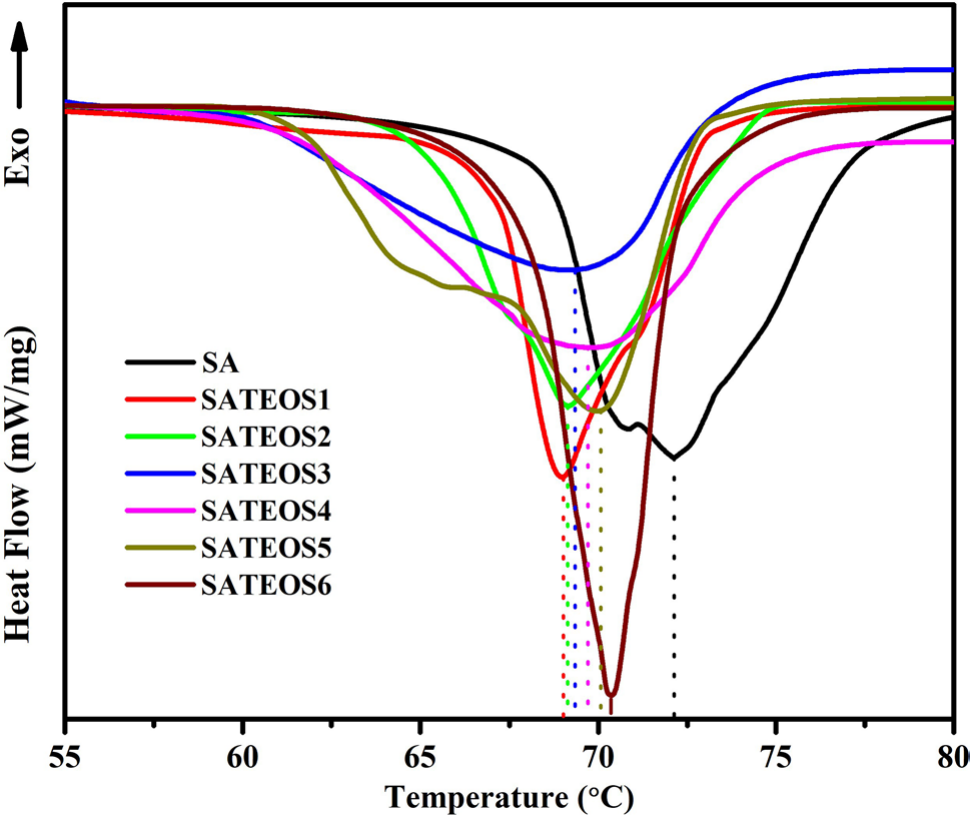
Melting curves of the SA and microencapsulated SA with SiO_2_ shell.

**Figure 11:**
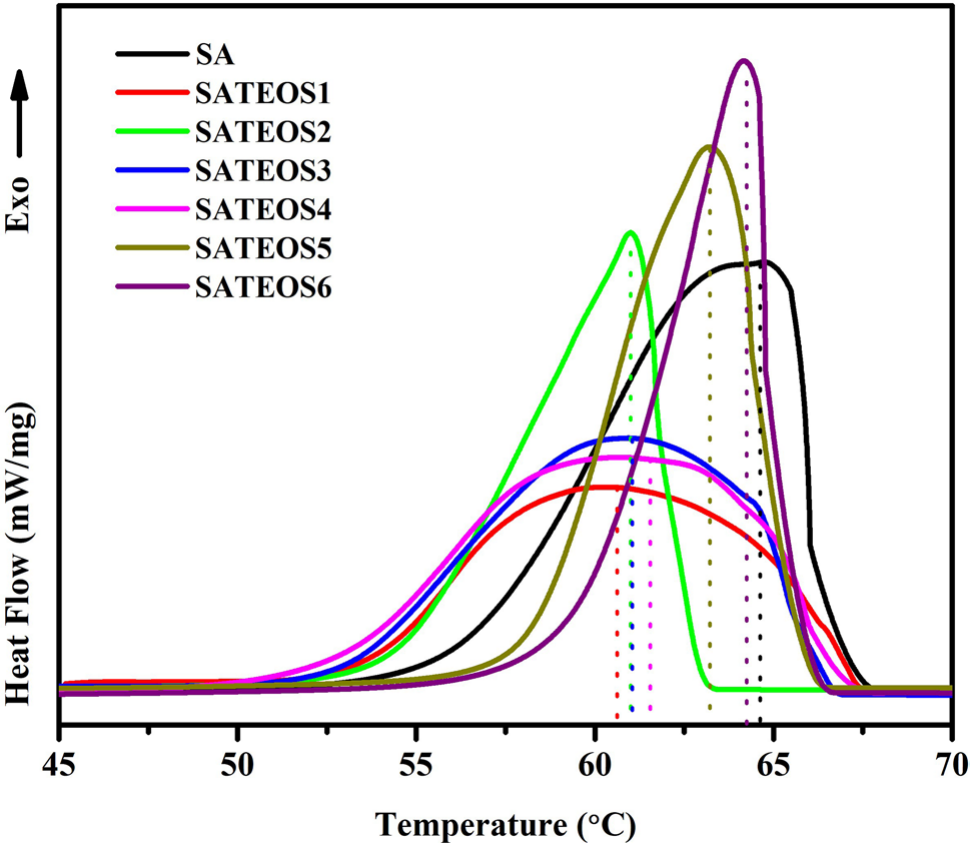
Solidifying curves of the SA and microencapsulated SA with SiO_2_ shell.

The melting and solidifying behaviour of the bulk SA along with microencapsulated SA with SiO_2_ shell are shown in Figs. 10 and 11, respectively and the thermal data are presented in Table 4. It is observed that melting and solidifying temperatures of microencapsulated SA are varying. Once the amount of SA is increased, the melting and solidifying temperature is increased and reach near to the values of bulk SA. After microencapsulation of SA, the silica wall enhances the crystallisation temperature where its wall act as nucleus in promoting the heterogeneity. Therefore, as the SA amount is increased, the melting (Fig. 10) and solidifying (Fig. 11) temperatures are also increased gradually^49,51,64^. Among all microencapsulated SA samples, SATEOS6 exhibits the highest melting and solidifying temperature followed by SATEOS5, SATEOS4, SATEOS3, SATEOS2 and SATEOS1.

**Table 4:** DSC data of the SA and microencapsulated SA.

Sample ID	Melting		Solidifying		Encapsulation ratio (%)	Encapsulation efficiency (%)
	Temperature (°C)	Latent heat (J/g)	Temperature (°C)	Latent heat (J/g)		
SA	72.09	200.90	64.65	194.42	–	–
SATEOS1	68.97	144.42	60.60	123.14	71.89	67.68
SATEOS2	69.16	148.17	60.99	127.53	73.75	69.74
SATEOS3	69.31	157.97	61.02	136.86	78.63	74.58
SATEOS4	69.65	160.42	61.49	142.62	79.85	76.66
SATEOS5	69.96	174.15	63.16	150.28	86.68	82.07
SATEOS6	70.37	182.53	64.27	160.12	90.86	86.68

SATEOS1 shows the lowest melting (68.97 °C) and solidifying (60.60 °C) temperature attributed to the lower in particle size where the motion of the SA particles within the microcapsule is very less as well as SiO_2_ shell forms thick layer, thus, the core material limits the extension and movement^49^. This assumption is correlated with the SEM result where SATEOS1 shows smaller particles size (Fig. 9b) which correlates that SA molecule is confined in very small area of microcapsule. The temperature difference between melting and solidification of bulk as well as all microencapsulated SA with SiO_2_ shell exhibit in the range of 6.10–8.37 °C. This result suggests that microencapsulated SA can be used as a potential energy storage materials owing to the good thermal conductivity of SiO_2_ shell^65^.

It is seen from Table 4 that SATEOS6 has highest enthalpy among all microencapsulated SA owing to the proper encapsulation as observed by SEM (Fig. 9g). The encapsulation ratio of SA can be calculated from Eq. (1) by comparing the latent heat data of the microencapsulated SA^49^.

1 $$\text{R}\%= \frac{\Delta\text{H}_{{\rm MEPCM,m}}}{\Delta\text{H}_{{\rm PCM,m}}} \times 100$$

The value of R is the encapsulation ratio (%) of the microencapsulated SA, ΔH_MEPCM,m_ represents the melting latent heat of the microencapsulated SA, and ΔH_PCM,m_ represents the melting latent heat of the SA. Besides, the encapsulation efficiency (%) was computed as another important technical parameters as shown in Eq. (2)^49^.

2 $$\text{E}\%= \frac{\Delta\text{H}_{{\rm MEPCM,m}}+ \Delta\text{H}_{{\rm MEPCM,s}}}{\Delta\text{H}_{{\rm PCM,m}}+\Delta\text{H}_{{\rm PCM,s}}} \times 100$$

The value of E represents the encapsulation efficiency (%) of the microencapsulated SA, ΔH_MEPCM,s_ is the solidifying latent heat of the microencapsulated SA, and ΔH_PCM,s_ represents the solidifying latent heat of the SA.

From Table 4, it is found that the encapsulation ratio and efficiency of SATEOS1 is found to be 71.89% and 67.68% whereas SATEOS6 exhibits 90.86% and 86.68% (Table 4), respectively. SATEOS6 samples has exhibited highest encapsulation ratio and efficiency amongst all microencapsulated SA inferring that it has high heat storage capacity. Therefore, it requires high energy to transform from solid to liquid phase. In addition, during cooling process, the difference between melting and solidifying temperature in all microencapsulated SA and bulk SA infer that silica shell is space-confined during the synthesis of microencapsulation. Thus result suggests that as the amount of SA is increased, the encapsulation ratio and efficiency gradually increased (Table 4).

### Thermal stability of the microencapsulated SA with SiO_2_ shell

**Figure 12:**
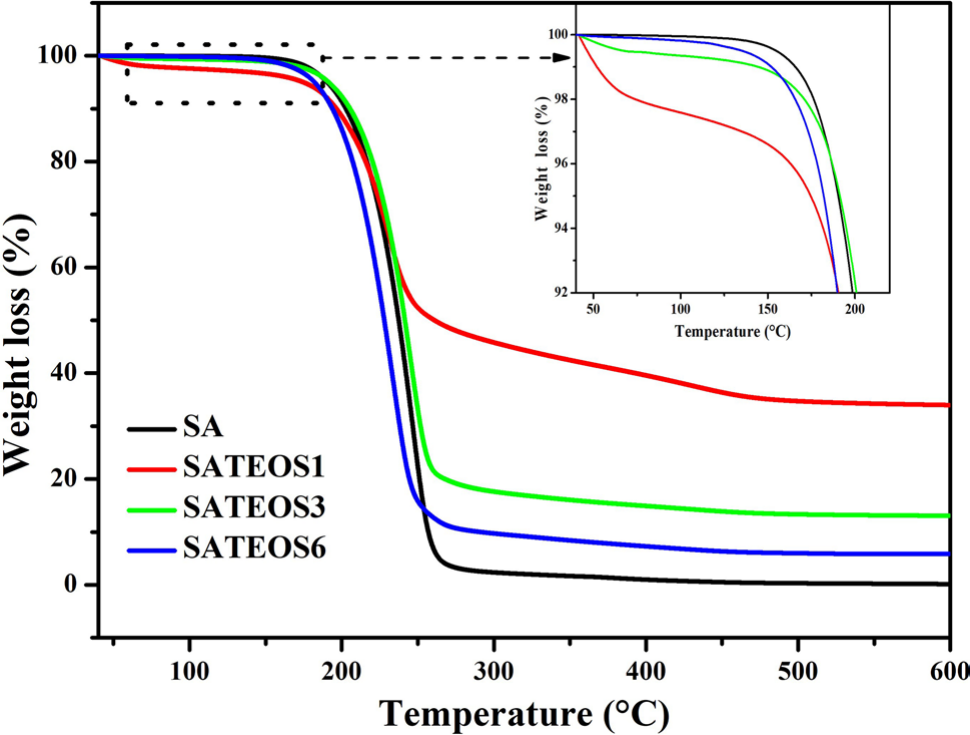
TGA curves of the SA, SATEOS1, SATEOS3 and SATEOS6.

The TGA curves of bulk SA and microencapsulated SA (SATEOS1, SATEOS3 and SATEOS6) with SiO_2_ shell are shown in Fig. 12. The thermal stability performance of bulk SA with microencapsulated samples i.e. SATEOS1, SATEOS3 and SATEOS6 has been compared. It can be seen from the TGA curves that bulk SA as well as microencapsulated SA exhibit smooth and a very slight decrease in weight loss starting from 40 to 190 °C. Up to this temperature, there is no thermal decomposition of bulk SA whereas the microencapsulated SA release the adsorbed water even after the samples were dried at 45 °C for 24 h. That has caused the slight weight loss^49^ but beyond this temperature, materials start to decompose. In lower amount of SA i.e. SATEOS1, the adsorbed water content was higher thus, the mass loss up to 190 °C is higher (inset of Fig. 12). Once the temperature is increased beyond 190 °C, the mass loss of samples is started to occur attributed to the decomposition process. The bulk SA is started to decompose from 190 °C and remain only 4% at 260 °C whereas SATEOS1, SATEOS3 and SATEOS6 at this temperature remained 50%, 20% and 12%, respectively. After 300 °C, it is observed that the weight loss of bulk SA is around 97.60% whereas SATEOS1, SATEOS3 and SATEOS6 exhibit around 54.20%, 82.40% and 90.30%, respectively. As the amount of SA increased, the SiO_2_ content decreased (Table 3) as well as thinning of shell was observed in SEM (Fig. 9). Therefore, the mass loss of the microencapsulated SA is lower compared to bulk SA attributed to the advantageous characteristics of SiO_2_ shell which encourages to form carbonaceous-silicate charred layer onto the SA surface that insulate the core SA and slow down the escape of volatile products generated during the thermal decomposition^10^. This charred layer creates a physical protective barrier which restrict the transfer of flammable molecule to the gas phase^66,67^. Apart from that, we can see significant results on the amount of weight loss where SATEOS1 exhibits lower value compared to SATEOS3, SATEOS6 and SA. This is due to the fact that the amount of SA in the SATEOS1 is lower where SiO_2_ shells form a thick layer than SATEOS3 and SATEOS6. On the contrary, the bulk SA demonstrates total weight loss up to 99.50% at 415 °C. However, SATEOS1, SATEOS3 and SATEOS6 exhibits 62.50%, 85.50% and 93.76% weight loss at 415 °C, respectively. This finding suggests that the addition of TEOS has improved the SA decomposition owing to the formation of SiO_2_ layers on the surface of SA. The layers may create a physical protective barrier thus, improvement in thermal stability of the microencapsulated SA is observed.

### Thermal reliability of the microencapsulated SA with SiO_2_ shell

**Figure 13:**
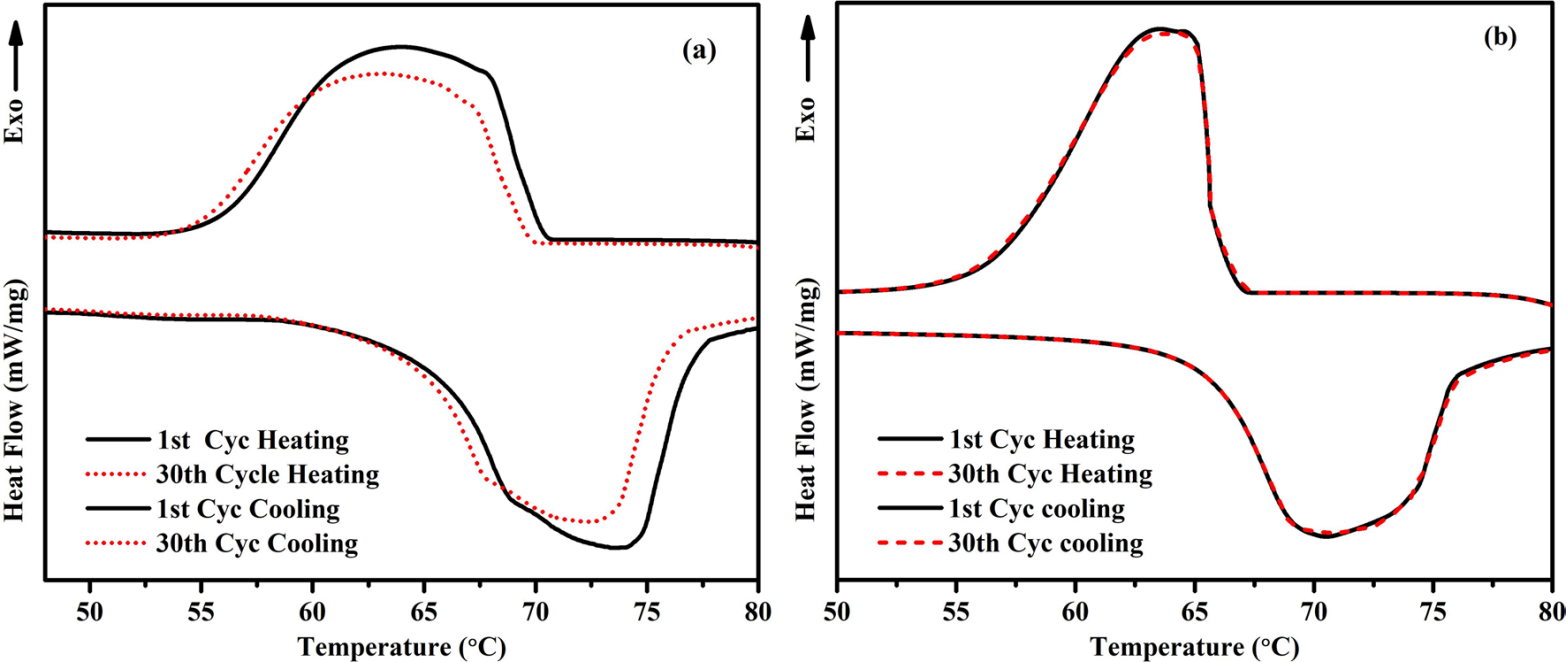
DSC curves of **(a)** SA, and **(b)** SATEOS6 samples obtained at 1st and 30th cycles of heating and cooling.

The thermal reliability results of bulk SA as well as the best sample among microencapsulated samples i.e. SATEOS 6 after 30 DSC heating and cooling cycles^51,52^ are shown in Fig. 13. It can be seen that bulk SA (Fig. 13a) exhibits shifting in melting/solidifying temperature as well as enthalpy value whereas SATEOS6 (Fig. 13b) does not show any difference in temperatures and enthalpy values even after 30th cycles of heating and cooling process. The bulk SA shows the melting temperature at 72.10 °C and solidifying temperature at 64.69 °C with 201.0 J/g and 194.10 J/g melting and solidifying enthalpy after the 1st cycle, respectively. After 30th cycles, these values are decreased up to 71.24 °C for melting and 63.53 °C for solidifying temperature whereas enthalpy values are decreased by 10%. The shifting in melting and solidifying temperatures and reduction in enthalpy values revealed that the bulk SA is not reliable for the application without microencapsulation. But, once the proper microencapsulation is occurred (SATEOS6), there is no change in melting and solidifying temperatures as well as in enthalpy values (Fig. 13b). After the microencapsulation with SiO_2_ shell, the SA can be used as PCM for thermal application especially in construction field owing to its optimum melting and solidifying temperatures as well as stable enthalpy values.

## Conclusions

In this research, systematic studies for microencapsulation of SA as core material and SiO_2_ as shell material was performed. TEOS was used as the precursor to form supporting and protective layers of SiO_2_ on the surface of SA. FT-IR, XRD, XPS, SEM and EDS results exhibited the presence of SiO_2_ after successful synthesis of microencapsulated SA. SEM analysis showed that SATEOS6 sample exhibited well-defined globular particle that was surrounded by the SiO_2_ shell on the surface of SA. However, MEPCM with lower amount of SA exhibited agglomerations which reduced the properties of PCM. XPS analysis exhibited the presence of Si–O–Si and Si–OH in microencapsulated samples which revealed about the adsorption of SiO_2_ on the surface of SA. According to the thermal properties analysis, SATEOS6 showed the most promising ability as thermal energy storage with a melting and solidifying temperatures at 70.37 °C and 64.27 °C as well as melting and solidifying latent heats of 182.53 J/g and 160.12 J/g, respectively. The maximum encapsulation efficiency was found to be at 86.68% for SATEOS6. TGA and thermal cycle analysis by DSC confirmed that SATEOS6 has good thermal stability and reliability even after 30th cycles of heating and cooling process.
